# Behavioral Patterns and Associations with Glucose Control During 12-Week Randomized Free-Living Clinical Trial of Day and Night Hybrid Closed-Loop Insulin Delivery in Adults with Type 1 Diabetes

**DOI:** 10.1089/dia.2016.0307

**Published:** 2017-07-01

**Authors:** Ali Emami, Malgorzata E. Willinska, Hood Thabit, Lalantha Leelarathna, Sara Hartnell, Sibylle Dellweg, Carsten Benesch, Julia K. Mader, Manuel Holzer, Harald Kojzar, Thomas R. Pieber, Sabine Arnolds, Mark L. Evans, Roman Hovorka

**Affiliations:** ^1^ Wellcome Trust-MRC Institute of Metabolic Science, University of Cambridge, Cambridge, United Kingdom.; ^2^ Department of Paediatrics, University of Cambridge, Cambridge, United Kingdom.; ^3^ Department of Diabetes & Endocrinology, Cambridge University Hospitals NHS Foundation Trust, Cambridge, United Kingdom.; ^4^ Central Manchester University Hospitals NHS Foundation Trust and University of Manchester, Manchester, United Kingdom.; ^5^ Profil, Neuss, Germany.; ^6^ Division of Endocrinology and Diabetology, Department of Internal Medicine, Medical University of Graz, Graz, Austria.

**Keywords:** Closed-loop systems, Type 1 diabetes, Behavior, Meals, Insulin boluses

## Abstract

***Objectives:*** We evaluated patterns of meal intake, insulin bolus delivery, and fingerstick glucose measurements during hybrid closed-loop and sensor-augmented pump (SAP) therapy, including associations with glucose control.

***Methods:*** Data were retrospectively analyzed from pump-treated adults with type 1 diabetes who underwent, in random order, 12 weeks free-living closed-loop (*n* = 32) and 12 weeks SAP (*n* = 33) periods. We quantified daily patterns of main meals, snacks, prandial insulin boluses, correction boluses, and fingerstick glucose measurements by analyzing data recorded on the study glucometer and on study insulin pump.

***Results:*** We analyzed 1942 closed-loop days and 2530 SAP days. The total number of insulin boluses was reduced during closed-loop versus SAP periods by mean 1.0 per day (95% confidence interval 0.6–1.4, *P* < 0.001) mainly because of a reduced number of correction boluses by mean 0.7 per day (0.4–1.0, *P* < 0.001). Other behavioral patterns were unchanged. The carbohydrate content of snacks but not the number of snacks was positively correlated with (1) glycemic variability as measured by standard deviation of sensor glucose (closed-loop *P* < 0.05; SAP *P* < 0.01), (2) mean sensor glucose (*P* < 0.05), and (3) postintervention HbA1c (*P* < 0.05). Behavioral patterns explained 47% of between-subject variance in glucose variability during SAP period and 30%–33% of variance of means sensor glucose and postintervention HbA1c.

***Conclusion:*** Fewer correction boluses are delivered during closed-loop period. The size of snacks appears to worsen glucose control possibly because of carbohydrate-rich content of snacks. Modifiable behavioral patterns may be important determinants of glucose control.

## Introduction

Over the past decades, advances in continuous glucose monitoring^1^ and insulin pump technologies^2^ have led to improved care and quality of life in people with type 1 diabetes. Although the accuracy and reliability of the devices have gradually increased,^1^ many users do not achieve glucose targets.^3^

Further improvements in outcomes may be achieved by linking sensor glucose measurements to insulin delivery such as when applying low glucose suspend^4^ or predictive glucose management approaches^5^ and ultimately closed-loop glucose control (the “artificial pancreas”), the latter characterized by automated graduated insulin delivery by insulin pump *below* and *above* preprogramed level directed by a control algorithm. Two main configurations of the closed-loop control system, the single-hormone (insulin-only) and the dual-hormone systems (i.e., addition of glucagon), have been shown to offer tighter glucose control than current treatment modalities.^6^ Given the delayed absorption of current subcutaneously delivered rapid-acting insulin analogues, “hybrid closed-loop systems” benefiting from user-initiated prandial insulin boluses appear more appropriate than the fully closed-loop approach.^7^ Within the hybrid closed-loop approach, user interactions may affect outcomes directly such as when omitting prandial boluses or indirectly through changes in eating habits.

Randomized outpatient clinical trials of the hybrid closed-loop control system over up to a 3-month period have been performed^6^ such as that by Thabit et al. assessing the feasibility, safety, and efficacy of the 12-week home use of a single-hormone closed-loop control system in comparison with sensor-augmented pump therapy.^8^ The medium-term clinical trials provide an opportunity to enhance our understanding of behavioral patterns, their changes during the use of the closed-loop control, and associations with glycemic control. In this article, we performed a retrospective analysis of the data obtained by Thabit et al.^8^ to explore these trends.

## Research Design and Methods

### Study design and participants

We retrospectively analyzed data from an open-label, multicenter, randomized, crossover trial conducted under free-living home conditions in adults with type 1 diabetes in the United Kingdom, Germany, and Austria.^8^ Each participant used the hybrid closed-loop system and sensor-augmented pump therapy (open loop) for a 12-week period. Overall, 33 participants [18 males, 15 females; age (mean ± standard deviation (SD)), 40.0 ± 9.4 years; duration of insulin pump therapy, 7.8 ± 5.9 years; HbA1c at screening, 8.5% ± 0.7%; total daily insulin dose, 0.62 ± 0.15 U/(kg·d)] completed the 12-week period of sensor-augmented pump therapy, while 32 of those successfully completed the 12-week closed-loop period. During hybrid closed-loop and sensor-augmented pump periods, the participants applied bolus wizard to administer prandial boluses and were able to deliver correction boluses between meals on their own volition. Detailed information regarding the study design and results has been published.^8^

### Capture of meal, insulin delivery, and fingerstick glucose measurement data

During both periods, the participants used study insulin pump (Dana R Diabecare, Sooil, Seoul, South Korea) that stored insulin delivery data, including insulin boluses and meal data entered during the use of bolus wizard. The study glucometer (FreeStyle Navigator II; Abbott Diabetes Care, Alameda, CA) stored information about fingerstick glucose measurements and meals/snacks not accompanied by insulin bolus. Food diaries were not used.

### Data and statistical analysis

Daily (midnight to midnight) behavioral patterns evaluating eating, insulin bolusing, and glucose self-monitoring habits were determined from data downloaded from the study glucometer and study insulin pump, including the number and size (in grams of carbohydrates) of main meals per day (main meal defined as that containing 25 g carbohydrates or more per 70 kg body weight), the number and size of snacks per day (snack defined as that containing <25 g carbohydrates per 70 kg body weight), the number of insulin boluses per day, the number of small boluses per day (defined as <10% of average total daily dose), the number of correction boluses per day (defined as <10% of average total daily dose, delivered 15 min or more apart from a meal and at a glucose value at or >8 mmol/L), and the number of fingerstick glucose measurements per day. Mean sensor glucose, glucose variability as measured by the standard deviation of sensor glucose, and HbA1c at the end of study interventions assessed glucose control.

From the closed-loop intervention, we included in the analysis days during which closed loop was operational for at least 80% of the time to ensure that glucose control was mostly directed by the closed-loop system. During both study periods, days without meals were excluded from the analysis.

Behavioral patterns during closed-loop and sensor-augmented pump periods were compared using paired *t* test. Associations between behavioral patterns and glucose control were evaluated using the Pearson correlation coefficient. The linear regression analysis was used to quantify the amount of explained between-subject variance in glucose control endpoints as a function of behavioral patterns. The analyses should be considered hypotheses generating and no corrections for multiple comparisons have been made.

Data processing was performed using Matlab version 2013b (The MathWorks, Inc., Natick, MA) and the statistical analysis was performed using SPSS version 23 (IBM Software, Hampshire, UK). Data are reported as mean ± SD unless stated otherwise. A value of *P* < 0.05 was considered statistically significant.

## Results

The total number of days included in the analysis was 4472 (1942 closed-loop days, 2530 sensor-augmented pump days). We excluded 830 days when closed loop was operational for <80% of the time and also excluded 292 days with no meal data.

**FIG. 1.**
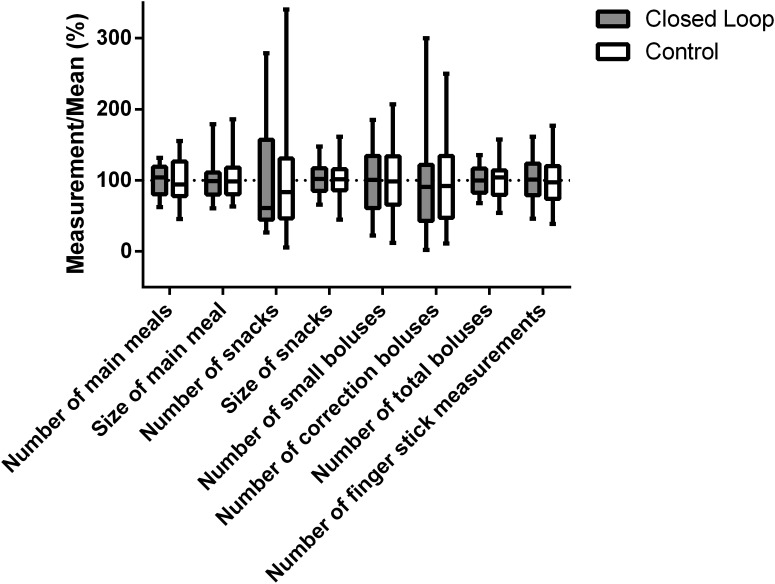
Distribution of behavioral patterns during closed-loop (*n* = 32) and control (*n* = 33) periods. Values are relative to population means (100%).

The distribution of behavioral patterns including eating habits, insulin boluses, and fingerstick measurements is shown in Figure 1. The number of snacks per day and the number of correction boluses per day displayed the highest relative variability among participants. Table 1 compares behavioral patterns between closed-loop and control periods. Eating habits were not affected by the use of closed loop with an identical number of reported meals and snacks, and no change in meal or the snack size. The number of fingerstick glucose measurements was also similar—the analysis combines calibrating and noncalibrating fingerstick glucose measurements. The total number of insulin boluses was reduced during closed-loop than during sensor-augmented pump therapy by mean 1.0 bolus per day [95% confidence interval (95% CI) 0.6–1.4; *P* < 0.001] mainly because of less frequent administration of correction boluses reduced by mean 0.7 bolus per day (95% CI 0.4–1.0; *P* < 0.001) so that around 0.6 correction boluses were administered per day during closed loop. Of these correction boluses, around 0.5 (0.484) were given during daytime (07:00–23:00) and around 0.1 (0.135) were given during night-time (23:00–07:00).

**Table 1.** Behavioral Patterns and Glucose Control During Closed-Loop and Control Periods

	*Closed-loop (*n* = 32)*	*Control (*n* = 33)*	*Paired difference (95% CI)*^a^	P
No. of main meals per day	2.9 ± 0.6	2.7 ± 0.8	−0.2 (−0.4 to 0.07)	0.166
Size of main meal per day (grams of CHO)	51.2 ± 12.3	52.7 ± 13.9	1.0 (−0.7 to 3.0)	0.243
No. of snacks per day	1.2 ± 0.9	1.1 ± 0.8	−0.1 (−0.3 to 0.1)	0.386
Size of snacks per day (grams of CHO)	19.2 ± 3.8	19.4 ± 4.4	0.004 (−0.8 to 0.8)	0.992
No. of boluses per day	4.4 ± 0.9	5.4 ± 1.5	1.0 (0.6 to 1.4)	<0.001
No. of small boluses per day^b^	2.4 ± 1.0	3.0 ± 1.5	0.7 (0.2 to 1.0)	0.003
No. of correction boluses per day^c^	0.6 ± 0.5	1.3 ± 0.9	0.7 (0.4 to 1.0)	<0.001
No. of fingerstick measurements per day	4.2 ± 1.3	4.3 ± 1.5	0.01 (−0.3 to 0.4)	0.950
Mean sensor glucose (mmol/L)^d^	8.7 ± 1.1	9.3 ± 1.6	0.6 (0.3 to 1.0)	<0.001
Glucose variability (SD, mmol/L)^d^	3.4 ± 0.7	3.6 ± 0.7	0.3 (0.1 to 0.4)	<0.001
Postintervention HbA1c (%)^d^	7.3 ± 0.8	7.6 ± 1.1	0.3 (0.1 to 0.5)	0.003

Mean ± SD values are reported.

^a^ Mean differences (closed-loop period minus control period), with 95% CIs for the mean.

^b^ Small boluses include those boluses that did not exceed 10% of the subject's average total daily dose.

^c^ Correction boluses include small boluses 15 min or more apart from a meal and delivered when the sensor glucose reading exceeded 8 mmol/L.

^d^ As reported previously.^8^

95% CI, 95% confidence interval; CHO, carbohydrates; SD, standard deviation.

Associations between behavioral patterns and glucose control are reported in Table 2. The increased size but not the number of snacks that an individual consumed was associated with deteriorated glucose control, including increased mean sensor glucose, glucose variability, and postintervention HbA1c. This applied to both closed-loop and sensor-augmented pump periods (*P* < 0.05) with a particular effect on glucose variability during sensor-augmented pump intervention (*P* < 0.01). Glucose variability was positively associated with the size of main meals during closed-loop period (*P* < 0.05). The number of fingerstick glucose measurements was associated with reduced glucose variability during sensor-augmented pump period (*P* < 0.05) but not during closed-loop period. No further significant associations were found. About one half (47%) of between-subject variance of glucose variability was explained by behavioral patterns during sensor-augmented pump period. For other glucose control end points, the linear regression model explained about one-third (30%–33%) of between-subject variance during closed-loop and sensor-augmented pump periods.

**Table 2.** Correlations Between Behavioral Patterns and Glucose During Closed-Loop and Control Periods

	*Closed-loop (*n* = 32)*			*Control (*n* = 33)*		
	*Mean glucose*	*Glucose variability*	*Postintervention HbA1c*	*Mean glucose*	*Glucose variability*	*Postintervention HbA1c*
No. of main meals per day	0.030	0.003	0.045	−0.06	−0.202	0.015
Size of main meal per day (grams of CHO)	0.306	0.377^*^	0.276	0.268	0.317	0.209
No. of snacks per day	0.077	−0.136	0.058	−0.147	−0.214	−0.088
Size of snacks per day (grams of CHO)	0.404^*^	0.365^*^	0.441^*^	0.388^*^	0.531^**^	0.428^*^
No. of boluses per day	−0.064	−0.221	0.031	−0.018	−0.172	−0.087
No. of small boluses per day	−0.050	−0.245	0.017	−0.181	−0.325	−0.229
No. of correction boluses per day	0.092	0.004	0.134	−0.015	−0.097	−0.106
No. of fingerstick measurements per day	−0.205	−0.270	−0.259	−0.278	−0.367^*^	−0.193
R^2^ of the linear regression model (%)^a^	31	30	31	32	47^*^	33

Pearson correlation coefficient is reported.

^*^ *P* < 0.05.

^**^ *P* < 0.01.

^a^ The linear regression model included all of the behavioral traits already described.

## Discussion

The results demonstrate that the number of correction boluses decreases during closed-loop period, reducing the total number of insulin boluses administered per day compared with sensor-augmented pump therapy reducing therapy burden. The size but not the number of snacks that an individual consumes is associated with poorer glycemic control as measured by mean glucose, glucose variability, and HbA1c during sensor-augmented pump and closed-loop interventions. The effect of snack size on glucose variability appears to be diminished during closed-loop intervention.

Other studies investigated associations between behavioral patterns and glycemic control. Pfützner et al.^9^ reported that pump users performing more frequent fingerstick glucose measurements have better glycemic control and lower glucose variability. This is consistent with our findings that during sensor-augmented pump therapy, the number of fingerstick measurements is associated with lower glucose variability, whereas displaying no such trend during closed-loop period. We determined that the combined effect of behavioral patterns during sensor-augmented pump therapy explains greater amount of glucose variability than when closed-loop period was applied (47% vs. 30%, respectively). Our results suggest that the use of a closed-loop system lessens the influence of behavioral patterns on glucose variability but not on mean glucose levels.

Data from T1D exchange^3^ registry showed that the frequency of self-monitoring of blood glucose is strongly correlated with lower HbA1c levels in all age groups even after adjusting for confounding factors such as insurance coverage, household income, and insulin pump therapy. In this study, HbA1c levels were not associated with frequency of self-monitoring of blood glucose that can be explained by the use of continuous glucose monitoring, which enables glucose values and the rate of glucose change to be freely inspected without resorting to fingerstick glucose measurements.

Delahanty and Halford^10^ examined self-reported diet-related behaviors during the Diabetes and Control and Complications Trial and reported that overtreating hypoglycemia and consuming extra snacks outside meal plan were associated with higher HbA1c levels. Øverby et al.^11^ examined the association between skipping meals and snacking events in children and adolescents and observed that those who skip meals and have more snacks have poorer glycemic control. Our results show that the carbohydrate content but not the number of snacks per day is the diet-attributable behavior that significantly affects glucose control and particularly glucose variability. This finding may be explained by the anticipated simple-carbohydrates-rich content of snacks as opposed to larger meals that are expected to contain complex carbohydrates and other nutrients, slowing down carbohydrate absorption. Further research is warranted to confirm our findings.

The strength of our study is the relatively long duration of the interventions and the randomized multicenter controlled design supporting generalizability. Insulin bolusing and fingerstick measurements were reliably downloaded from the glucometer and insulin pump. The study is limited by the self-reported nature of meal-related information, which may not be fully reliable. This is, in part, because of the common challenge that patients face with carbohydrate counting, and specifically the potential impact of limited carbohydrate accuracy during the use of closed-loop or sensor-augmented pump therapy. Some of the common challenges include unreported meals, under or over estimation of carbohydrates, and multiple reporting of the same carbohydrate intake. We did not correct *P* values for multiple comparisons, given the hypotheses generating nature of the investigations.

In conclusion, we document that although eating patterns and self-blood glucose monitoring do not depend on the method of therapy, individuals using the closed-loop period show a decrease in the number of correction boluses. The size of snacks appears to worsen glucose control, possibly because of carbohydrate-rich content of snacks.

## References

[1] Rodbard D: Continuous glucose monitoring: a review of successes, challenges, and opportunities. Diabetes Technol Ther 2016;18(Suppl 2):S3–S13 10.1089/dia.2015.0417 PMC4717493 26784127

[2] Pickup JC: Banting memorial lecture 2014* technology and diabetes care: appropriate and personalized. Diabet Med 2015;32:3–13 25345658 10.1111/dme.12613

[3] Miller KM, Foster NC, Beck RW, et al.: T1D exchange clinic network: current state of type 1 diabetes treatment in the U.S.: updated data from the T1D exchange clinic registry. Diabetes Care 2015;38:971–978 25998289 10.2337/dc15-0078

[4] Bergenstal RM, Klonoff DC, Garg SK, et al.: Threshold-based insulin-pump interruption for reduction of hypoglycemia. N Engl J Med 2013;369:224–232 23789889 10.1056/NEJMoa1303576

[5] Choudhary P, Olsen BS, Conget I, et al.: Hypoglycemia prevention and user acceptance of an insulin pump system with predictive low glucose management. Diabetes Technol Ther 2016;18:288–291 26907513 10.1089/dia.2015.0324 PMC4870649

[6] Thabit H, Hovorka R: Coming of age: the artificial pancreas for type 1 diabetes. Diabetologia 2016;59:1795–1805 27364997 10.1007/s00125-016-4022-4 PMC4969330

[7] Weinzimer SA, Steil GM, Swan KL, et al.: Fully automated closed-loop insulin delivery versus semiautomated hybrid control in pediatric patients with type 1 diabetes using an artificial pancreas. Diabetes Care 2008;31:934–939 18252903 10.2337/dc07-1967

[8] Thabit H, Martin T, Roman H, et al.: Home use of an artificial beta cell in type 1 diabetes. N Engl J Med 2015;373:2129–2140 26379095 10.1056/NEJMoa1509351 PMC4697362

[9] Pfützner A, Weissmann J, Mougiakakou S, et al.: Glycemic variability is associated with frequency of blood glucose testing and bolus: post hoc analysis results from the ProAct Study. Diabetes Technol Ther 2015;17:392–397 25734860 10.1089/dia.2014.0278 PMC4432784

[10] Delahanty LM, Halford BN: The role of diet behaviors in achieving improved glycemic control in intensively treated patients in the Diabetes Control and Complications Trial. Diabetes Care 1993;16:1453–1458 8299434 10.2337/diacare.16.11.1453

[11] Øverby N, Margeirsdottir H, Brunborg C, et al.: Sweets, snacking habits, and skipping meals in children and adolescents on intensive insulin treatment. Pediatr Diabetes 2008;9:393–400 18774998 10.1111/j.1399-5448.2008.00381.x

